# A series of sequences convergent to Euler’s constant

**DOI:** 10.1186/s13660-018-1727-6

**Published:** 2018-06-19

**Authors:** Li-Jiang Jia, Bin Ge, Li-Li Liu, Yi Ran

**Affiliations:** 1 School of Economics and Management, Harbin Engineering University, Harbin, P.R. China; 2 Department of Applied Mathematics, Harbin Engineering University, Harbin, P.R. China

**Keywords:** 11Y60, 40A05, 40A20, 41A25, 34E05, 35J70, Euler’s constant, Rate of convergence, Asymptotic expansion

## Abstract

In this paper, using continued fraction, we provide a new quicker sequence convergent to Euler’s constant. We demonstrate the superiority of our new convergent sequences over DeTemple’s sequence, Mortici’s sequences, Vernescu’s sequence, and Lu’s sequence.

## Introduction

As it is known, defining some new approximations toward fundamental constants plays an important role in the field of mathematical constants. One of the most famous constants is Euler’s constant $\gamma =0.577215\dots $, which is defined as the limit of the sequence

1.1 $$\begin{aligned}& \gamma_{n}=\sum_{k=1}^{n} \frac{1}{k} - \ln n \end{aligned}$$

and has numerous applications in many areas of pure and applied mathematics, such as analysis, number theory, theory of probability, applied statistics, and special functions.

Up until now, many authors have devoted great efforts and achieved much in the area of improving the convergence rate of the sequence $(\gamma_{n})_{n\geq 1}$. Among them, there are many inspiring achievements. For example, the estimate

1.2 $$\begin{aligned}& \frac{1}{2(n+1)}< \gamma_{n}-\gamma < \frac{1}{2n} \quad \mathrm{(Young)} \end{aligned}$$

was given in [1–4].

In [5, 6], a new sequence $(D_{n})_{n\geq 1}$ converging faster to *γ* was introduced, which is defined as

1.3 $$\begin{aligned}& D_{n}=1+\frac{1}{2}+\frac{1}{3}+\cdots + \frac{1}{n} - \ln \biggl(n+ \frac{1}{2} \biggr). \end{aligned}$$

DeTemple also concluded that the speed of the new sequence to *γ* is of order $n^{-2}$ since

1.4 $$\begin{aligned}& \frac{1}{24(n+1)^{2}}< D_{n}-\gamma < \frac{1}{24n^{2}} \quad \mathrm{(DeTemple)}. \end{aligned}$$

Another modification was provided by Vernescu [7] as

1.5 $$\begin{aligned}& V_{n}=1+\frac{1}{2}+\frac{1}{3}+\cdots + \frac{1}{n-1}+\frac{1}{2n}- \ln n, \end{aligned}$$

who proved that

1.6 $$\begin{aligned}& \frac{1}{12(n+1)^{2}}< \gamma -V_{n}< \frac{1}{12n^{2}}. \end{aligned}$$

It is easy to conclude that though (1.3) and (1.5) only make slight modifications on the Euler’s sequence (1.1), but the convergent rates are significantly improved from $n^{-1}$ to $n^{-2}$.

Moreover, Mortici obtained some sequences converging even faster than (1.1), (1.3), and (1.5). More specifically, Mortici [8] constructed the following two sequences:

1.7 $$\begin{aligned}& u_{n}=1+\frac{1}{2}+\frac{1}{3}+\cdots + \frac{1}{n-1}+\frac{1}{(6-2 \sqrt{6})n}- \ln \biggl(n+\frac{1}{\sqrt{6}} \biggr), \end{aligned}$$

1.8 $$\begin{aligned}& v_{n}=1+\frac{1}{2}+\frac{1}{3}+\cdots + \frac{1}{n-1}+\frac{1}{(6+2 \sqrt{6})n}- \ln \biggl(n-\frac{1}{\sqrt{6}} \biggr). \end{aligned}$$

Both (1.7) and (1.8) had been proved to converge to *γ* as $n^{-3}$.

Moreover, Mortici [9] introduced the following class of sequences:

1.9 $$\begin{aligned}& \mu_{n}(a,b)=\sum_{k=1}^{n} \frac{1}{k} +\ln \bigl(e^{a/(n+b)}-1\bigr)- \ln a, \end{aligned}$$

where $a,b\in \mathbb{R}$, $a>0$. They proved that, among the sequences $(\mu_{n}(a,b))_{n\geq 1}$, in the case of $a=\sqrt{2}/2$ and $b=(2+\sqrt{2})/4$ the privileged sequence offers the best approximations of *γ* since

1.10 $$\begin{aligned}& \lim_{n\rightarrow \infty }n^{3} \biggl( \mu_{n} \biggl(\frac{ \sqrt{2}}{2},\frac{2+\sqrt{2}}{4} \biggr)-\gamma \biggr)=\frac{ \sqrt{2}}{96}. \end{aligned}$$

Recently, Lu, Song, and Yu [10] provided some approximations of Euler’s constant. A new important sequence was defined as follows:

1.11 $$\begin{aligned}& \gamma_{n,k}^{(s)}=1+\frac{1}{2}+ \frac{1}{3}+\cdots +\frac{1}{n}- \ln n - \frac{1}{k}\ln \biggl(1+\frac{a_{1}}{n+\frac{a_{2}n}{n+\frac{a _{3}n}{n+\frac{a_{4}n}{n+\ddots +a_{s}}}}} \biggr), \end{aligned}$$

where

$$\begin{aligned}& a_{1}=\frac{k}{2}, \qquad a_{2}=\frac{2-3k}{12}, \qquad a_{3}= \frac{3k^{2}+4}{12(3k-2)}, \\& a_{4}=-\frac{15k^{4}-30k^{3}+60k^{2}-104k+96}{20(3k-2)(3k ^{2}+4)},\qquad \dots. \end{aligned}$$

Two particular sequences were provided as

1.12 $$\begin{aligned}& \gamma_{n,1}^{(2)}=1+\frac{1}{2}+\frac{1}{3}+ \cdots +\frac{1}{n}- \ln n - \ln \biggl(1+\frac{a_{1}}{n+a_{2}} \biggr), \end{aligned}$$

1.13 $$\begin{aligned}& \gamma_{n,2}^{(3)}=1+\frac{1}{2}+\frac{1}{3}+ \cdots +\frac{1}{n}- \ln n - \frac{1}{2}\ln \biggl(1+ \frac{a_{1}}{n+\frac{a_{2}n}{n+a_{3}}} \biggr). \end{aligned}$$

These two sequences converge faster than all other sequences mentioned since for all $n\in \mathbb{N}$,

$$\frac{7}{288(n+1)^{3}}< \gamma -\gamma_{n,1}^{(2)}< \frac{7}{288n ^{3}} \quad \mbox{and} \quad \frac{1}{180(n+1)^{4}}< \gamma - \gamma_{n,2}^{(3)}< \frac{1}{180(n-1)^{4}}. $$

On the other hand, Lu [11] introduced the following class of sequences:

1.14 $$\begin{aligned} K_{n,k}^{(s)} = &1+\frac{1}{2}+\frac{1}{3}+\cdots +\frac{1}{n}- \ln n \\ &{}- \frac{1}{k}\ln \biggl(1+\frac{a_{1}}{n}+\frac{a_{2}}{n^{2}}+ \frac{a _{3}}{n^{3}}+\cdots +\frac{a_{s}}{n^{s}} \biggr), \end{aligned}$$

where $k,s\in \mathbb{N}$. They also proved that, among the sequences $(K_{n,k}^{(s)})_{n\geq 1}$, in the case of

$$\begin{aligned}& a_{1}=\frac{k}{2}, \qquad a_{2}=\frac{k(3k-2)}{24}, \qquad a_{3}= \frac{k^{2}(k-2)}{48}, \\& a_{4}=\frac{k(15k^{3}-60k^{2}+20k+48)}{5760},\qquad \dots, \end{aligned}$$

the privileged sequence offers the best approximations of *γ* since when $s=1$,

1.15 $$\begin{aligned}& \lim_{n\rightarrow \infty }n^{2}\bigl(K_{n,k}^{(1)}- \gamma\bigr)= \frac{3k-2}{24}; \end{aligned}$$

when $s=2$,

1.16 $$\begin{aligned}& \lim_{n\rightarrow \infty }n^{3}\bigl(K_{n,k}^{(2)}- \gamma\bigr)=\frac{k ^{2}-2k}{48}; \end{aligned}$$

when $s=3$,

1.17 $$\begin{aligned}& \lim_{n\rightarrow \infty }n^{4}\bigl(K_{n,k}^{(3)}- \gamma\bigr)=\frac{15k ^{3}-60k^{2}+20k+48}{5760}. \end{aligned}$$

These works motivated our study. In this paper, our main goal is to modify the sequence based on the early works of DeTemple, Moritici, and Lu and provide a new convergent sequence of relatively simple form with higher speed.

The rest of this paper is arranged as follows. In Sect. 2, we provide the main results and, in Sect. 3, we prove them.

## The main results

### Lemma 2.1

*For any fixed* $a,b\in \mathbb{R}$, *we have the following convergent sequence for Euler’s constant*:

2.1 $$\begin{aligned}& N_{n,a,b}=1+\frac{1}{2}+\frac{1}{3}+\cdots + \frac{1}{n-1}+ \frac{1}{an}- \ln (n+b). \end{aligned}$$

*Moreover*, *for* $a=1$ *and* $b=0$, *we have*

2.2 $$\begin{aligned}& \lim_{n\rightarrow \infty }n(N_{(n,1,0)}-\gamma)= \frac{1}{2}; \end{aligned}$$

*for* $a=1$ *and* $b=1/2$, *we have*

2.3 $$\begin{aligned}& \lim_{n\rightarrow \infty }n^{2}(N_{(n,1,\frac{1}{2})}- \gamma)=\frac{1}{24}; \end{aligned}$$

*for* $a=2$ *and* $b=0$, *we have*

2.4 $$\begin{aligned}& \lim_{n\rightarrow \infty }n^{2}(N_{(n,2,0)}- \gamma)= \frac{1}{24}; \end{aligned}$$

*for* $a=6-2\sqrt{6}$ *and* $b={1}/{\sqrt{6}}$, *we have*

2.5 $$\begin{aligned}& \lim_{n\rightarrow \infty }n^{3}(N_{(n,6-2\sqrt{6},\frac{1}{ \sqrt{6}})}- \gamma)=-\frac{1}{18\sqrt{6}}; \end{aligned}$$

*and for* $a=6+2\sqrt{6}$ *and* $b=-{1}/{\sqrt{6}}$, *we have*

2.6 $$\begin{aligned}& \lim_{n\rightarrow \infty }n^{3}(N_{(n,6+2\sqrt{6},-\frac{1}{ \sqrt{6}})}- \gamma)=\frac{1}{18\sqrt{6}}. \end{aligned}$$

Using Lemma 2.1, we have the following conclusion.

### Corollary 2.2

*The fastest possible sequence* $(N_{n,a,b})_{n\geq 1}$ *is obtained only for*

$$\textstyle\begin{cases} \frac{1}{a}-b-\frac{1}{2}=0,\\ -\frac{1}{a}+b^{2}+b+ \frac{1}{3}=0,\end{cases} $$

*and*

2.7 $$\begin{aligned}& \lim_{n\rightarrow \infty }n^{3}(N_{n,a,b}- \gamma)= \frac{1}{3} \biggl(\frac{1}{a}-b^{3}- \frac{3b^{3}}{2}-b- \frac{1}{4} \biggr). \end{aligned}$$

### Theorem 2.3

*For any fixed* $s\in \mathbb{N}$, *there exist* $k\in \mathbb{N}$ *and* $a, b \in \mathbb{R}$ *such that the following sequence converges to Euler’s constant*:

2.8 $$\begin{aligned} \gamma \approx \gamma_{n,k,a,b}^{(s)} =& 1+ \frac{1}{2}+\frac{1}{3}+ \cdots +\frac{1}{n-1}+ \frac{1}{an}- \ln (n+b) \\ &{}- \frac{1}{k}\ln \biggl(1+\frac{a_{1}}{n}+\frac{a_{2}}{n^{2}}+ \frac{a_{3}}{n^{3}}+ \cdots +\frac{a_{s}}{n^{s}} \biggr), \end{aligned}$$

*where*

$$\begin{aligned}& a_{1}=k \biggl(\frac{1}{a}-b-\frac{1}{2} \biggr), \\& a_{2}=-\frac{1}{2a}+\frac{b^{2}}{2}+ \frac{b}{2}+\frac{1}{6}+\frac{(2k-2abk-ak)^{2}}{8a^{2}k}+\frac{2k-2abk-ak}{4ak}, \\& a_{3}=\frac{k}{3a}-\frac{b^{3}k}{3}- \frac{b^{2}k}{2}- \frac{bk}{3}-\frac{k}{12}-\frac{a_{1}}{3}- \frac{a_{1}^{2}}{2}+a_{2}+a _{1}a_{2}- \frac{a_{1}^{3}}{3},\qquad \dots. \end{aligned}$$

*Furthermore*, *let*

2.9 $$\begin{aligned}& \gamma_{n,k,a,b}^{(1)}=1+\frac{1}{2}+ \frac{1}{3} +\cdots + \frac{1}{n-1}+\frac{1}{an}- \ln (n+b)- \frac{1}{k}\ln \biggl(1+\frac{a _{1}}{n} \biggr), \end{aligned}$$

2.10 $$\begin{aligned}& \gamma_{n,k,a,b}^{(2)}=1+ \frac{1}{2}+ \frac{1}{3}+\cdots + \frac{1}{n-1}+\frac{1}{an}- \ln (n+b)- \frac{1}{k}\ln \biggl(1+\frac{a _{1}}{n}+\frac{a_{2}}{n^{2}} \biggr), \end{aligned}$$

2.11 $$\begin{aligned}& \gamma_{n,k,a,b}^{(3)}=1+ \frac{1}{2}+ \frac{1}{3}+\cdots + \frac{1}{n-1}+\frac{1}{an}- \ln (n+b)- \frac{1}{k}\ln \biggl(1+\frac{a _{1}}{n}+\frac{a_{2}}{n^{2}}+ \frac{a_{3}}{n^{3}} \biggr). \end{aligned}$$

*Then we also have*, *for* $s=1$,

2.12 $$\begin{aligned}& \lim_{n\rightarrow \infty }n^{3} \bigl( \gamma_{n,k,a,b}^{(1)}- \gamma \bigr) \\& \quad =\frac{4k-4ab^{3}k-6ab^{2}k-4abk-ak-4aa_{1}-6aa _{1}^{2}-4aa_{1}^{3}}{12ak}; \end{aligned}$$

*for* $s=2$,

2.13 $$\begin{aligned}& \lim_{n\rightarrow \infty }n^{4} \bigl( \gamma_{n,k,a,b}^{(2)}- \gamma \bigr)=-\frac{1}{4a}+ \frac{b^{4}}{4}+\frac{b^{2}}{2}+ \frac{b}{4}+\frac{1}{20}; \end{aligned}$$

*and for* $s=3$,

2.14 $$\begin{aligned} \lim_{n\rightarrow \infty }n^{5}\bigl( \gamma_{n,a,b,k}^{(3)}- \gamma\bigr) = &\frac{1}{5a}- \frac{b^{5}}{5}-\frac{b^{4}}{2}-\frac{2b ^{3}}{3}-\frac{b^{2}}{2}- \frac{b}{5}-\frac{1}{30}- \frac{a_{1}}{5k} \\ &{}+\frac{a_{2}}{k}-\frac{3a_{3}}{5k}+\frac{2a_{1}a _{2}}{k}+ \frac{a_{2}a_{3}}{k}-\frac{2a_{1}a_{3}}{k} \\ &{}-\frac{a_{1} ^{2}a_{3}}{k}-\frac{a_{1}a_{2}^{2}}{k}+\frac{2a_{1}^{2}a_{2}}{k}+ \frac{a _{1}^{3}a_{2}}{k}-\frac{a_{1}^{4}}{2k} \\ &{}-\frac{2a_{1}^{3}}{3k}-\frac{4a _{1}^{5}}{25}. \end{aligned}$$

### Lemma 2.4

*If* $(x_{n})_{n\geq 1}$ *converges to zero and there exists the limit*

2.15 $$\begin{aligned}& \lim_{n\rightarrow \infty }n^{s}(x_{n}-x_{n+1})=l \in [- \infty,+\infty ] \end{aligned}$$

*with* $s>1$, *then*

2.16 $$\begin{aligned}& \lim_{n\rightarrow \infty }n^{s-1}x_{n}= \frac{l}{s-1}. \end{aligned}$$

Lemma 2.4 was first proved by Moritici [12]. From Lemma 2.4 we can see that the speed of convergence of the sequence $(x_{n})_{n\geq 1}$ increases together with the value *s* satisfying (2.15).

## The proof of Theorem 2.3

Based on the argument of Theorem 2.1 in [13] or Theorem 5 in [14], we need to find the value of $a_{1}\in \mathbb{R} $ that produces the most accurate approximation of the form

3.1 $$\begin{aligned}& N_{n,a,b}=1+\frac{1}{2}+\frac{1}{3}+\cdots + \frac{1}{n-1}+ \frac{1}{an}- \ln (n+b). \end{aligned}$$

To measure the accuracy of this approximation, a method is to say that an approximation (3.1) is better as $N_{n,a,b}-\gamma $ faster converges to zero. Using (3.1), we have

3.2 $$\begin{aligned} N_{n,a,b}-N_{n+1,a,b} = &-\frac{1}{n}+ \frac{1}{an}-\frac{1}{a(n+1)} \\ &{}- \ln (n+b)+\ln (n+1+b). \end{aligned}$$

Developing in power series in $1/n$, we have

3.3 $$\begin{aligned} N_{n,a,b}-N_{n+1,a,b} = & \biggl(\frac{1}{a}-b- \frac{1}{2} \biggr)\frac{1}{n ^{2}}+ \biggl(-\frac{1}{a}+b^{2}+b+ \frac{1}{3} \biggr)\frac{1}{n^{3}} \\ &{}+ \biggl(\frac{1}{a}-b^{3}- \frac{3b^{2}}{2}-b-\frac{1}{4} \biggr)\frac{1}{n^{4}} \\ &{}+ \biggl(-\frac{1}{a}+b^{4}+2b^{3}+2b ^{2}+b+1 \biggr)\frac{1}{n^{5}}+O \biggl( \frac{1}{n^{6}} \biggr). \end{aligned}$$

From Lemma 2.4 we know that the speed of convergence of the sequence $(N_{n,a,b})_{n\geq 1}$ is even higher than the value *s* satisfying (2.15). Thus, using Lemma 2.4, we have:

(i) If $\frac{1}{a}-b-\frac{1}{2}\neq 0 $, then the convergence rate of the sequence $(N_{n,a,b}-\gamma)_{n \geq 1}$ is $1/n$ since
  $$\lim_{n\rightarrow \infty }n(N_{n,a,b}-\gamma)= \frac{1}{a}-b- \frac{1}{2}\neq 0. $$
(ii) If $\frac{1}{a}-b-\frac{1}{2}=0 $, then from (3.3) we have
  $$\begin{aligned} N_{n,a,b}-N_{n+1,a,b} = & \biggl(-\frac{1}{a}+b^{2}+b+ \frac{1}{3} \biggr)\frac{1}{n^{3}}+ \biggl(\frac{1}{a}-b^{3}- \frac{3b^{2}}{2}-b- \frac{1}{4} \biggr)\frac{1}{n^{4}} \\ &{}+ \biggl(-\frac{1}{a}+b^{4}+2b ^{3}+2b^{2}+b+1 \biggr) \frac{1}{n^{5}}+O \biggl(\frac{1}{n^{6}} \biggr). \end{aligned}$$

If $-\frac{1}{a}+b^{2}+b+\frac{1}{3}\neq 0 $, then the rate of convergence of the sequence $(N_{n,a,b}-\gamma)_{n \geq 1}$ is $n^{-2}$ since

$$\lim_{n\rightarrow \infty }n^{2}(N_{n,a,b}- \gamma)=- \frac{1}{2a}+\frac{b^{2}}{2}+\frac{b}{2}+ \frac{1}{6}. $$

If $-\frac{1}{a}+b^{2}+b+\frac{1}{3}=0 $, then from (3.3) we have

$$\begin{aligned} N_{n,a,b}-N_{n+1,a,b} = & \biggl(\frac{1}{a}-b^{3}- \frac{3b^{2}}{2}-b- \frac{1}{4} \biggr)\frac{1}{n^{4}} \\ &{}+ \biggl(-\frac{1}{a}+b^{4}+2b ^{3}+2b^{2}+b+1 \biggr) \frac{1}{n^{5}} +O \biggl(\frac{1}{n^{6}} \biggr), \end{aligned}$$

and the rate of convergence of the sequence $(N_{n,a,b}-\gamma)_{n \geq 1}$ is $n^{-3}$ since

$$\lim_{n\rightarrow \infty }n^{3}(N_{n,a,b}- \gamma)= \frac{1}{3a}-\frac{b^{3}}{3}-\frac{b^{2}}{2}- \frac{b}{3}- \frac{1}{12}. $$

Moreover, for $a=1$ and $b=0$, we have

3.4 $$\begin{aligned}& \lim_{n\rightarrow \infty }n(N_{(n,1,0)}-\gamma)= \frac{1}{2}; \end{aligned}$$

for $a=1$ and $b=1/2$, we have

3.5 $$\begin{aligned}& \lim_{n\rightarrow \infty }n^{2}(N_{(n,1,\frac{1}{2})}- \gamma)=\frac{1}{24}; \end{aligned}$$

for $a=2$ and $b=0$, we have

3.6 $$\begin{aligned}& \lim_{n\rightarrow \infty }n^{2}(N_{(n,2,0)}- \gamma)= \frac{1}{24}; \end{aligned}$$

for $a=6-2\sqrt{6}$ and $b={1}/{\sqrt{6}}$, we have

3.7 $$\begin{aligned}& \lim_{n\rightarrow \infty }n^{3}(N_{(n,6-2\sqrt{6},\frac{1}{ \sqrt{6}})}- \gamma)=-\frac{1}{18\sqrt{6}}; \end{aligned}$$

and for $a=6+2\sqrt{6}$ and $b=-{1}/{\sqrt{6}}$, we have

3.8 $$\begin{aligned}& \lim_{n\rightarrow \infty }n^{3}(N_{(n,6+2\sqrt{6},-\frac{1}{ \sqrt{6}})}- \gamma)=\frac{1}{18\sqrt{6}}. \end{aligned}$$

### Proof of Theorem 2.3

We define the sequence $(\gamma_{n,a,b,k}^{(s)})_{n\geq 1}$ by the relations

3.9 $$\begin{aligned} \gamma \approx \gamma_{n,k,a,b}^{(s)} = &1+ \frac{1}{2}+\frac{1}{3}+ \cdots +\frac{1}{n-1}+ \frac{1}{an}- \ln (n+b) \\ &{}- \frac{1}{k}\ln \biggl(1+\frac{a_{1}}{n}+\frac{a_{2}}{n^{2}}+ \frac{a_{3}}{n^{3}}+ \cdots +\frac{a_{s}}{n^{s}} \biggr) \end{aligned}$$

and

3.10 $$\begin{aligned} \gamma \approx \gamma_{n,k,a,b}^{(1)} = &1+ \frac{1}{2}+\frac{1}{3}+ \cdots +\frac{1}{n-1}+ \frac{1}{an}- \ln (n+b) \\ &{}- \frac{1}{k}\ln \biggl(1+\frac{a_{1}}{n} \biggr). \end{aligned}$$

Using a similar method as in (3.1)–(3.3), we have

3.11 $$\begin{aligned}& \gamma_{n,k,a,b}^{(1)}-\gamma_{n+1,k,a,b}^{(1)} \\& \quad =\frac{-3k+3ab ^{2}k+3abk+ak+3aa_{1}+3aa_{1}^{2}}{3akn^{3}} \\& \qquad {}+\frac{2k-2abk-ak-2aa _{1}}{2akn^{2}} \\& \qquad {}+\frac{4k-4ab^{3}k-6ab^{2}k-4abk-ak-4aa _{1}-6aa_{1}^{2}-4aa_{1}^{3}}{4akn^{4}} \\& \qquad {}+\frac{-5k+5ab^{4}k+10ab ^{3}k+10ab^{2}k+5abk+ak-5aa_{1}+10aa_{1}^{2}}{5akn^{5}} \\& \qquad {}+\frac{10aa _{1}^{2}+10aa_{1}^{3}+5aa_{1}^{4}}{5akn^{5}}+O \biggl(\frac{1}{n^{6}} \biggr). \end{aligned}$$

The fastest possible sequence $(\gamma_{n,a,b,k}^{(1)})_{n\geq 1}$ is obtained when

$$\textstyle\begin{cases} \frac{2k-2abk-ak-2aa_{1}}{2ak}=0, \\ \frac{-3k+3ab^{2}k+3abk+ak+3aa _{1}+3aa_{1}^{2}}{3ak}=0.\end{cases} $$

Then we have

$$\begin{aligned}& \lim_{n\rightarrow \infty }n^{3}\bigl( \gamma_{n,a,b,k}^{(1)}- \gamma\bigr) \\& \quad =\frac{4k-4ab^{3}k-6ab^{2}k-4abk-ak-4aa_{1}-6aa_{1} ^{2}-4aa_{1}^{3}}{12ak}, \end{aligned}$$

and the rate of convergence is $n^{-3}$.

For example, for $a=2$ and $b=1/(2\sqrt{3})$,

$$\begin{aligned}& \lim_{n\rightarrow \infty }n^{3}\bigl( \gamma_{n,2,\frac{1}{2\sqrt{3}},k}^{(1)}-\gamma\bigr)=\frac{-k+3 \sqrt{3}k+3\sqrt{3}k^{2}-k^{3}}{72\sqrt{3}k}, \end{aligned}$$

and the rate of convergence is $n^{-3}$.

Next, we define the second sequence with the previous conclusions:

3.12 $$\begin{aligned} \gamma_{n,k,a,b}^{(2)} = &1+\frac{1}{2}+ \frac{1}{3}+\cdots + \frac{1}{n-1}+\frac{1}{an}- \ln (n+b) \\ &{}- \frac{1}{k}\ln \biggl(1+\frac{a _{1}}{n}+\frac{a_{2}}{n^{2}} \biggr), \end{aligned}$$

where $a_{1}=\frac{2k-2abk-ak}{2a} $.

Then we get the equation

3.13 $$\begin{aligned}& \gamma_{n,k,a,b}^{(2)}-\gamma_{n+1,k,a,b}^{(2)} \\& \quad = \biggl(- \frac{1}{a}+b^{2}+b+ \frac{1}{3}+\frac{a_{1}^{2}}{k}+ \frac{a_{1}}{k}-\frac{2a_{2}}{k} \biggr)\frac{1}{n^{3}} \\& \qquad {}+ \biggl( \frac{1}{a}-b^{3}- \frac{3b^{2}}{2}-b-\frac{1}{4}-\frac{3a_{1} ^{2}}{2k}+\frac{3a_{1}a_{2}}{k} \biggr)\frac{1}{n^{4}} \\& \qquad {}+ \biggl(-\frac{a _{1}^{3}}{k}-\frac{a_{1}}{k}+\frac{3a_{2}}{k} \biggr)\frac{1}{n^{4}}+ \biggl(-\frac{1}{a}+b^{4}+2b^{3} \\& \qquad {}+2b^{2}+b+\frac{1}{5}+ \frac{a _{1}}{k}-\frac{4a_{2}}{k}+\frac{2a_{1}^{2}}{k}+\frac{2a_{2}^{2}}{k} \\& \qquad {}-\frac{6a_{1}a_{2}}{k}+\frac{2a_{1}^{3}}{k}- \frac{4a_{1}^{2}a_{2}}{k} + \frac{a_{1}^{4}}{k} \biggr)\frac{1}{n^{5}} +O \biggl(\frac{1}{n^{6}} \biggr). \end{aligned}$$

Taking

$$\textstyle\begin{cases} a_{1}=\frac{k}{a}-bk-\frac{k}{2}, \\ a_{2}=-\frac{k}{2a}+\frac{b ^{2}k}{2}+\frac{bk}{2}+\frac{k}{6}+\frac{a_{1}^{2}}{2}+ \frac{a_{1}}{2},\\ \frac{1}{a}-b^{3}-\frac{3b^{2}}{2}-b- \frac{1}{4}-\frac{3a_{1}^{2}}{2k}+\frac{3a_{1}a_{2}}{k}-\frac{a_{1} ^{3}}{k}-\frac{a_{1}}{k}+\frac{3a_{2}}{k}=0, \end{cases} $$

we obtain the fastest sequence $(\gamma_{n,a,b,k}^{(2)})_{n\geq 1}$ with convergent rate $n^{-4}$ since

$$\begin{aligned} \lim_{n\rightarrow \infty }n^{4}\bigl( \gamma_{n,a,b,k}^{(2)}- \gamma\bigr) = &-\frac{1}{4a}+ \frac{b^{4}}{4}+\frac{b^{3}}{2}+\frac{2b ^{2}}{4}+\frac{b}{4}+ \frac{1}{20}+\frac{a_{1}}{4k}-\frac{a_{2}}{k}+\frac{a _{1}^{2}}{2k} \\ &{}+\frac{a_{2}^{2}}{2k}-\frac{3a_{1}a_{2}}{2k}+\frac{a _{1}^{3}}{2k} - \frac{a_{1}^{2}a_{2}}{k} +\frac{a_{1}^{4}}{4k}. \end{aligned}$$

Moreover, for

$$\textstyle\begin{cases} a_{1}=\frac{k}{a}-bk-\frac{k}{2},\\ a_{2}=-\frac{k}{2a}+\frac{b^{2}k}{2}+\frac{bk}{2}+\frac{k}{6}+\frac{a_{1}^{2}}{2}+ \frac{a_{1}}{2}, \end{cases} $$

we define the third sequence with the previous conclusions:

3.14 $$\begin{aligned} \gamma_{n,k,a,b}^{(3)} = &1+\frac{1}{2}+ \frac{1}{3}+\cdots + \frac{1}{n-1}+\frac{1}{an}- \ln (n+b) - \frac{1}{k}\ln \biggl(1+\frac{a _{1}}{n}+\frac{a_{2}}{n^{2}}+ \frac{a_{3}}{n^{3}} \biggr). \end{aligned}$$

Then we have the equality

3.15 $$\begin{aligned}& \gamma_{n,k,a,b}^{(3)}-\gamma_{n+1,k,a,b}^{(3)} \\& \quad = \biggl(\frac{1}{a}-b ^{3}- \frac{3b^{2}}{2}-b-\frac{1}{4}-\frac{a_{1}}{k}-\frac{3a _{1}^{2}}{2k}+ \frac{3a_{2}}{k}-\frac{3a_{3}}{k}+\frac{3a_{1}a_{2}}{k}-\frac{a_{1}^{3}}{k} \biggr) \\& \qquad {}\frac{1}{n^{4}}+ \biggl(- \frac{1}{a}+b ^{4}+2b^{3}+2b^{2}+b+ \frac{1}{5}+\frac{a_{1}}{k}-\frac{4a _{2}}{k} \\& \qquad {}+\frac{2a_{2}^{2}}{k}+\frac{6a_{3}}{k}+ \frac{2a_{1}^{2}}{k}+ \frac{2a_{1}^{3}}{k}-\frac{6a_{1}a_{2}}{k}+\frac{4a _{1}a_{3}}{k}-\frac{4a_{1}^{2}a_{2}}{k}+ \frac{a_{1}^{4}}{k} \biggr)\frac{1}{n ^{5}} \\& \qquad {}+ \biggl(\frac{1}{a}-b^{5}- \frac{5b^{4}}{2}-\frac{10b ^{3}}{3}-\frac{5b^{2}}{2}-b-\frac{1}{6}- \frac{a_{1}}{k}+\frac{5a _{2}}{k}-\frac{3a_{3}}{k} \\& \qquad {} +\frac{10a_{1}a_{2}}{k}+\frac{5a_{2}a _{3}}{k}-\frac{10a_{1}a_{3}}{k}- \frac{5a_{1}^{2}a_{3}}{k}-\frac{5a _{1}a_{2}^{2}}{k}+\frac{10a_{1}^{2}a_{2}}{k} \\& \qquad {}+\frac{5a_{1}^{3}a _{2}}{k}-\frac{5a_{1}^{4}}{2k}-\frac{10a_{1}^{3}}{3k}- \frac{4a_{1} ^{5}}{5} \biggr)\frac{1}{n^{6}}+O \biggl(\frac{1}{n^{7}} \biggr). \end{aligned}$$

Taking

$$\textstyle\begin{cases} a_{3}=\frac{k}{3a}-\frac{b^{3}k}{3}-\frac{b^{2}k}{2}- \frac{bk}{3}-\frac{k}{12}-\frac{a_{1}}{3}-\frac{a_{1}^{2}}{2}+a_{2}+a _{1}a_{2}-\frac{a_{1}^{3}}{3}, \\ -\frac{1}{a}+b^{4}+2b^{3}+2b ^{2}+b+\frac{1}{5}+\frac{a_{1}}{k}-\frac{4a_{2}}{k}+\frac{2a_{2} ^{2}}{k}+\frac{6a_{3}}{k}+\frac{2a_{1}^{2}}{k}+\frac{2a_{1}^{3}}{k}, \\ -\frac{6a_{1}a_{2}}{k}+\frac{4a_{1}a_{3}}{k}-\frac{4a_{1}^{2}a _{2}}{k}+\frac{a_{1}^{4}}{k}=0, \end{cases} $$

we obtain the fastest sequence $(\gamma_{n,a,b,k}^{(3)})_{n\geq 1}$ with convergent rate $n^{-5}$ since

$$\begin{aligned}& \lim_{n\rightarrow \infty }n^{5}\bigl( \gamma_{n,a,b,k}^{(3)}- \gamma\bigr) \\& \quad =\frac{1}{5a}-\frac{b^{5}}{5}-\frac{b^{4}}{2}- \frac{2b ^{3}}{3}-\frac{b^{2}}{2}-\frac{b}{5}-\frac{1}{30}- \frac{a_{1}}{5k}+\frac{a_{2}}{k}-\frac{3a_{3}}{5k}+ \frac{2a_{1}a_{2}}{k}+ \frac{a_{2}a_{3}}{k} \\& \qquad {}-\frac{2a_{1}a_{3}}{k}-\frac{a _{1}^{2}a_{3}}{k}-\frac{a_{1}a_{2}^{2}}{k}+ \frac{2a_{1}^{2}a_{2}}{k}+\frac{a _{1}^{3}a_{2}}{k}-\frac{a_{1}^{4}}{2k}-\frac{2a_{1}^{3}}{3k}- \frac{4a _{1}^{5}}{25}. \end{aligned}$$

 □
